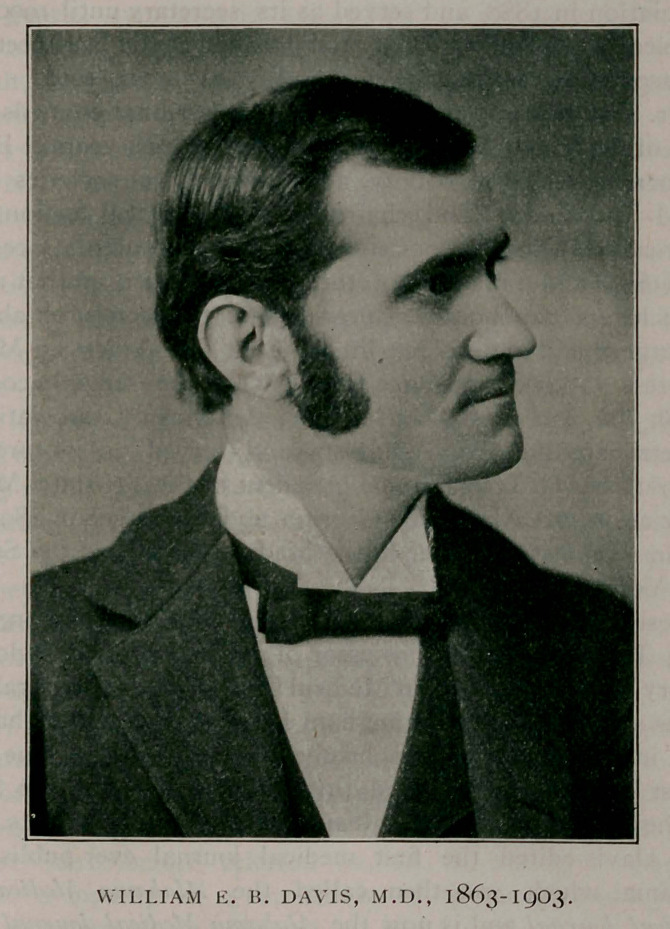# Dr. William E. B. Davis

**Published:** 1903-03

**Authors:** 


DR. WILLIAM E. B. DAVIS, of Birmingham, Ala., was killed at a railway crossing in that city February 24, 1903, presumably in attending to his professional work, though the JOURNAL is in ignorance of the details of the accident. This tragic news has caused a shock to the medical profession throughout the North American continent, greater than any similar event in years, for it is doubtful if any physician forty years old was as well known throughout the length and breadth of the land as this brilliant young surgeon. It is difficult to express appropriately or in fitting terms the deep sense of sorrow that fifteen years of intimate friendship engender, while we are yet enveloped in the pall of the awful tragedy that so mercilessly swept our friend into the realms of the hereafter. And it is even more

WILLIAM E. B. DAVIS, M.D., 1863-1903.

difficult to justly estimate the great virtues of a character so strong, so sweet, and so altogether lovely as was that of him on whose passing bier we lay this garland of affection.

William Elias Brownlee Davis, was born at Trussville, Jefferson County, Ala., November 25, 1863. His father was Dr. Elias Davis and his grandfather was Dr. Daniel Davis, both physicians of repute. He received his preliminary education at Trussville, studied medicine with his brother, Dr. John

D. S. Davis, and received his doctorate degree at Bellevue Hospital Medical College in 1884. He became the partner of his brother after graduation and so continued until his death. He spent a period in medical study in Europe soon after graduation, and on his return to Birmingham began his life work in the field of gynecology. He organised the Southern Surgical and Gynecological Association in 1888, and served as its secretary until 1900. He was elected president in 1901, and presided at the last meeting of the association, held at Cincinnati, in November, 1902.

Dr. Davis has been active in the professional councils of the State of Alabama and of the Nation for fifteen years. Besides membership in city, county, and state medical societies he has served as secretary and chairman of the surgical section of the American Medical Association, and has also been a vice-president (1893) and a member of the judicial council in that association; he was an honorary president of the section on abdominal surgery and gynecology in the First Pan-American Medical Congress (1893), and vice-president of the second congress (1896); he was a Fellow of the American Association of Obstetricians and Gynecologists and served as its president in 1901; he also served as president of the Tri-state Medical Association of Alabama, Georgia and Tennessee in 1891, and was an honorary member of the Medical Society of the State of New York.

Besides his membership in these and other medical organisations, Dr. Davis was professor of gynecology and abdominal surgery in the Birmingham Medical College, and for several years he was surgeon to the Birmingham Hospital of United Charities. Also, in association with his brother, he has conducted one of the largest and most successful private infirmaries in the South, for the treatment of surgical and gynecological patients. The Drs. Davis edited the first medical journal ever published in Alabama, which was then called the *Alabama Medical and Surgical Journal* and is now the *Alabama Medical Journal.*

Dr. Davis was a surgeon of original methods, advanced thought, and skilful technic. He was an acknowledged authority on the surgery of the liver, gall-bladder and ducts, and expected to publish a work embracing that field at an early day. He has conducted experiments for years upon these and similar subjects; his practice was exacting, and his patients in large numbers were drawn from the far distant regions of the South, being attracted by his superior skill and judgment. He and his brother were like one man with four hands at the operating table, it being rarely necessary to speak a word to each other, each having such a complete understanding of the other's technic and wishes.

In all that goes to make a resourceful, skilful and successful surgeon, Dr. Davis was specially gifted; he was at once modest, firm, gentle, sympathetic and, withal, so strong in his convictions of duty, and knew so well what to do, that it would be difficult to say that any man was his superior as an operator, or more endowed with that kind of judgment which often creates success in the midst of defeat, that wrings victory from disaster, and which gives an assurance of confidence in the face of what would seem utter hopelessness to many.

Taken hence in the midst of his greatest usefulness, in the mid-noon of a well-equipped manhood, it is difficult to say what this young man of forty summers would have accomplished had he been permitted to live out the full period of life expectation; but it is quite true that he has accomplished as much in his short life as most men do in a third longer time, and that it would have been next to impossible to achieve a higher claim to lasting renown had he lived to be eighty.

Among other accomplishments Dr. Davis was gifted as a speaker; his arguments were logical, his manner forceful, and his rhetoric sometimes ornate. Those who heard his eloquent after-dinner speech at the banquet of the American Association of Obstetricians and Gynecologists, at Washington last September, will not soon forget it.

Dr. Davis was married in August, 1897, and came to Niagara Falls and Buffalo with his charming bride, finally going East for the completion of the honeymoon journey. The surviving relatives are the widow, two sweet little girl children, and a brother, hereinbefore referred to, all of whom receive the assured sympathy of a multitude of warm personal friends throughout the whole country.

## Figures and Tables

**Figure f1:**